# Screening and GC-MS profiling of ethanolic extract of *Tylophora pauciflora*

**DOI:** 10.6026/97320630015425

**Published:** 2019-06-15

**Authors:** Thangarajan Starlin, Poochi Saravana Prabha, Basanth Kumar Allen Thayakumar, Velliyur Kanniappan Gopalakrishnan

**Affiliations:** 1 Department of Biochemistry and Bioinformatics, Karpagam Academy of Higher Education, Coimbatore, Tamilnadu, India 641 02; 2 Cancer Biology Department, Mitra RxDx Inc. Woburn MA 01801, USA

**Keywords:** Tylophora pauciflora, ethanolic extract, GC-MS analysis, bioactive compounds

## Abstract

Medicinal plants are boundless source of raw materials for the pharmaceutical. Identification of natural compounds from medicinal plant is helpful in the discovery of novel therapeutic agents. Tylophora pauciflora is a medicinal plant, which possess many biological activities such as antioxidant activity, anti-inflammatory activity and anti cancer activity. There is no GC-MS analysis reported on this plant. Thus, the present study is aimed to identify the present of phyto-chemical compounds from ethanolic extract of Tylophora pauciflora using GC-MS analysis. Results, the extract possess totally 14 bioactive compounds among that natural compound of n-hexadecanoic acid has highest % peak area and it have the variety of biological activities such as; anti-oxidant, 5-alpha-reductase-inhibitor, anti-fibrinolytic, hemolytic, antimicrobial activity, hypo-cholesterolemic, nematicide, pesticide, anti-androgenic flavor and hemolytic. It is concluded that the ethanolic extract of Tylophora pauciflora have biologically active compounds. In future by isolating and identifying, these compounds can be considered to treat the human disorders.

## Background

Medicinal plants are used in traditional treatments to cure variety of diseases. Modern medicine has evolved from folk medicines that use plant as a source of drugs 1. Currently 80% of the world population depends on plant-derived drugs for their beneficial effects to human because of its fewer side effects 2 and it contains numerous compounds with significant pharmacological potential, many of which may serve as lead compounds in the development of new drugs 3. Plants are rich in a wide variety of secondary metabolites such as tannins, terpenoids, alkaloids, and flavonoids etc., which have several biological properties 4. Consequently, Screenings of active compounds from natural sources has become relatively simpler and have played a major part in the development of new drugs from medicinal plants, which have efficient protection and treatment roles against various diseases including cancer and Alzheimer's diseases 5. Hence the development of effective methods to discover bioactive compounds from natural sources is deemed necessary. Several medicinal plants still available in the nature are not been investigated for their medicinal potential 6. The phyto-chemical compounds are not only encouraging for discovery of therapeutic prospective, but also have an active role towards invention of novel semi-synthetic and synthetic compounds 7. The screening of plant extracts is a novel strategy to find therapeutically active compounds in many plant species. Gas Chromatography-Mass Spectroscopy (GC/MS) combined analytical techniques to find the presence of phyto-chemical compounds from the plant extract 8.

Tylophora pauciflora is one of the vital medicinal plant belongs to the family of Asclepiadaceae and it is native of India and Southeast Asia. Tylophora genus has been used in the Ayurveda system for the treatment of various diseases. It is mainly used for bronchitis and bronchial asthma as a traditional medicine among the rural and tribal people of Odisha 9. In this genus, inflammation, antitumor, immunomodulatory, antioxidant, anti-asthmatic, anti-allergic properties smooth muscle relaxant, antihistaminic, hypotensive, analgesic, anticonvulsant, anti-rheumatic anti-inflammatory antiarthritis and anti-lupus in vivo activities are reported 10. In previous investigation, based on FTIR, EDX analysis Tylophora pauciflora contains Carboxylic acid; ester, alkane, lipids and minerals respectively 11. Hence, the objective of the present study is to identify the presence of phyto constituents in the ethanolic extract of Tylophora pauciflora using GC-MS technique.

## Methodology

### Plant collection and authentication:

The whole plant of Tylophora pauciflora was collected from the natural habitats, Tirunelveli district, Tamil Nadu, India and authenticated by Dr. C. Kalidass, Botanical survey of India, TNAU Campus, Coimbatore. The plant sample was collected and deposited in the Herbarium of the Botany Department, Bharathiar University, Coimbatore, Tamil Nadu. The voucher number is 006155. Fresh plant material was washed under running tap water, tipped on slain overnight, air dried and powdered.

### Extraction preparation:

A 100 g sample of dried plant powder was extracted in 500 ml of ethanol in an orbitory shaker for 72 hours. Repeated extraction was done with the same solvent until a clear colorless solvent was obtained. Obtained extract was evaporated to dryness and stored at 4°C in an airtight container for further use.

### GC-MS analysis:

GC-MS analyses of ethanolic extract were performed using a Thermo GC - Trace ultra Ver: 5.0 Thermo MS DSQ II systems and Gas chromatograph interfaced to a Mass spectrometer (GC-MS) equipped with DB 5 - MS capillary standard non-polar column (30mmX0.25mm 1D X 1 µMdf). For GC-MS detection, an electron ionization system with ionizing energy of 70 eV was used. Helium gas (99.999%) was used as the carrier gas at constant flow rate 1ml/min and an injection volume of 1µl was employed (split ratio of 10:1); Injector temperature 80°C; Ion-source temperature 250°C. The oven temperature was programmed from 70°C (isothermal for 2 min.), with an increase of 6°C/min, to 260°C. Mass spectra were taken at 70 eV; a scan interval of 0.5seconds and fragments from 50 to 650 Da. Total GC running time was 25 minutes. The relative % amount of each component was calculated by comparing its average peak area to the total areas, software adopted to handle mass spectra and chromatograms was a Turbo mass 12.

### Identification of bioactive compounds:

The identification of components was based on Willey and NIST libraries as well as comparison of their retention indices. The constituents were identified after comparison with those available in the computer library (NIST and Willey) 13 attached to the GCMS instrument and the results obtained. The name, molecular weight and the structure of the components of the test materials were ascertained, the relative percentage composition of each component was calculated by comparing its average peak area to the total area and tabulated.

## Result and Discussion:

**Figure 1:**
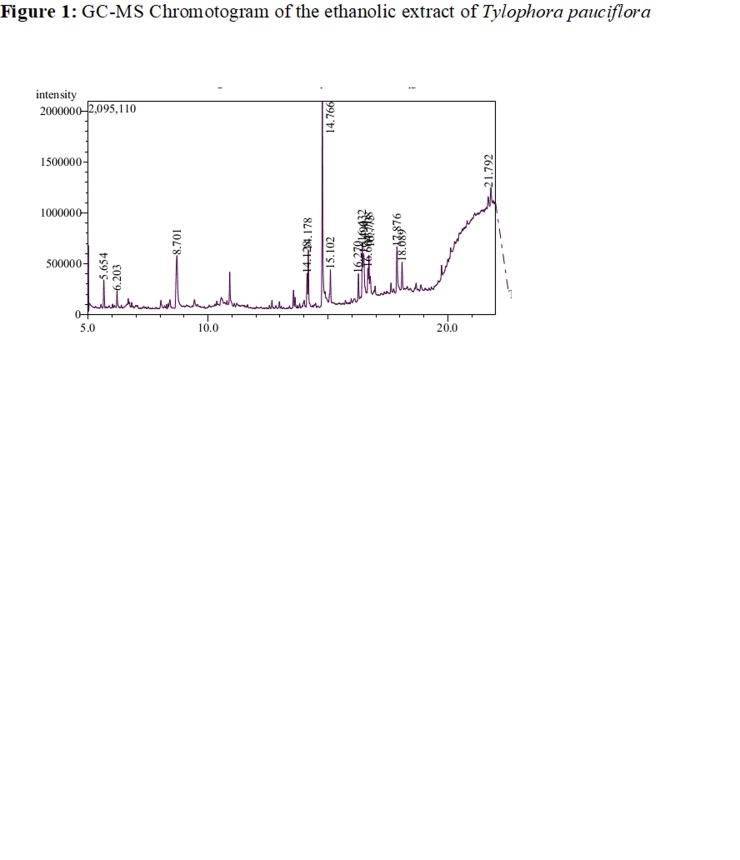
GC-MS Chromotogram of the ethanolic extract of Tylophora pauciflora

Gas chromatography separates the components of the mixture, and mass spectroscopy analyzes each of the components separately 14- 17. It is one of the best technique to identify bioactive constituents like long chain hydrocarbons, alcohols, acids, ester, alkaloids, steroids, amino and nitro compound etc. 18, 19. GC/MS is extensively applied in drug detection, environmental analysis, explosives investigation, medical, pharmaceutical, environmental, forensic applications and identification of unknown compounds of plants 20-22. Recent investigation are involved in the identification and isolation of new therapeutic compounds of medicinal importance from higher plants for specific diseases 23. Through the GCMS analysis, totally 14 natural compounds were identified in the ethanolic extract of Tylophora pauciflora. The active principles with their retention time (RT), molecular formula, molecular weight (MW) and concentration (%) in the ethanolic extract of Tylophora pauciflora are presented in Figure 1 and Table 1.

The n-hexa decanoic acid (28.31%) is is predominant followed by 9, 12-Octadecadienoic acid (Z, Z) (7.93%), 2-Nonenoic acid, methyl ester (7.67%), xanthosine (7.10%), beta-k-strophanthin (6.43%), 9, 12, 15-octadecatrienoic acid (6.14 %). Among the identified phyto compounds of the natural compound of n-Hexadecanoic acid has highest % peak area and it have the property of anti-oxidant, 5- alpha-reductase-inhibitor, anti-fibrinolytic, hemolytic, antimicrobial activity, hypo cholesterolemic nematicide, pesticide, antiandrogenic flavor and hemolytic 24-26. Octa deca dienoic acid have the property of anti-inflammatory, hypo cholesterolemic and anti-arthritic as reported by the earlier worker 27, 28. 9, 12, 15- Octadecatrienoic acid has anti-inflammatory, cancer preventive, hepato protective, Antioxidant and hypo cholesterolemic 29. The source of alpha-linolenic acid has been positively associated with prostate cancer. Alpha-linolenic acid from plant sources, such as flaxseed, does not affect prostate cancer risk. Based on the literature data these entire compound could effective contribute to the biological activity.

## Conclusion

The existence of various bioactive compounds in the Tylophora pauciflora validates the use of whole plant for various ailments by traditional specialists. However, isolation of individual phyto chemical constituents and subjecting it to the biological activity will definitely give rich results. From the results, it is concluded that Tylophora pauciflora contains various bioactive compounds. Therefore, it is recommended as a plant of phyto pharmaceutical importance.

## Conflict of Interest

The authors declare that there is no conflict of interests regarding the publication of this paper.

## Figures and Tables

**Table 1:** Components detected in the ethanolic extract of Tylophora pauciflora

S. No	RT	Name of the Compound	Molecular formula	MW	Peak Area %
1	5.654	Bicyclo(0.3.5)deca-1,3,5,7,9-pentaene	C10H8	128	3.96
2	6.203	1,2,3-Propanetriol, monoacetate	C5H10O4	134	2.19
3	8.701	Xanthosine	C10H12N4O6	284	7.1
4	14.178	2-Nonenoic acid, methyl ester	C10H18O2	170	7.67
5	14.766	n-Hexadecanoic acid	C16H32O2	256	28.31
6	15.102	Heptadecanoic acid methyl ester	C18H36O2	284	4.6
7	16.27	3,7,11,15-tetramethyl-2-hexadecen-1-ol	C20H40O	296	3.71
8	16.432	9,12-Octadecadienoic acid (Z,Z)	C18H34O2	294	7.93
9	16.49	9,12,15-Octadecatrienoic acid (ZZZ)	C19H32O2	292	6.14
10	16.664	Octadecanoic acid	C18H36O2	284	3.78
11	16.708	n-propyl 9,12- octadecadienoate linoleate	C18H32O2	280	5.5
12	16.775	Ethyl (9Z,12Z)-octadeca-9,12-dienoate	C20H36O2	308	1.89
13	17.876	beta-k-Strophanthin	C23H34O6	406	6.43
14	18.089	Methyl 2,4-di-O-acetyl-3,6-di-O-ethyl-	C15 H28O7	320	3.9
